# The impact of smart manufacturing demonstration projects on green innovation of Chinese firms: Based on random forest methods

**DOI:** 10.1016/j.heliyon.2024.e28925

**Published:** 2024-03-30

**Authors:** Jun Yang, Weihao Wang, Chunheng Fu, Xiaohui Xu, Qiuzhen Li

**Affiliations:** a School of Economics and Management, Zhejiang Sci-Tech University, 310018, China; b Yuanpei College, Shaoxing University, 312000, China

**Keywords:** Smart manufacturing demonstration projects, Green innovation, Random forest method, Information processing capability

## Abstract

Employing the data of Chinese A-share listed firms from 2010 to 2020 and random forest approaches, this paper investigates whether and how smart manufacturing demonstration projects influence green innovation of firms. The main results are as follows. First, smart manufacturing demonstration projects contribute to promoting firms' green innovation. Additionally, information processing capability improvement, innovation efficiency enhancement, public attention increasement, and signal effect are the main channels that improve firms' green innovation. Finally, the positive effect of smart manufacturing demonstration projects on firms’ green innovation is pronounced for capital-intensive firms, and firms in western and eastern regions.

## 1 Introduction

Green innovation is a primary way to solve environmental dilemma. In China, firms face challenges in promoting green innovation due to the imperfect market environment [1]. On the one hand, the innovation behavior of Chinese enterprises can not be separated from the drive of government subsidies and market policies [2]. Enterprises increase their R & D investment around subsidies and policies, while enterprises have less initiative innovation for development purposes, there are pitfalls in passive innovation. On the other hand, due to the unavailability of data, Information asymmetry and other reasons, enterprises lag behind in production technology and information input, showing a higher level of automation and lower level of intelligence, the green transformation of enterprises is blocked. As a result, there are few green innovation activities in Chinese companies [3]. Considering the “dual carbon” strategic goal, studying the key drivers of firms’ green innovation is crucial.

Information technologies such as AI, blockchains, cloud computing, and big data are booming, and smart manufacturing is becoming an important strategic direction. In terms of China, since 2015, the smart manufacturing pilot demonstration projects have been issued to promote manufacturing industry transformation [4]. Meanwhile, to successfully run those projects, the government has taken a series of security measures such as increasing financial support and improving services. Hence, if firms are listed in smart manufacturing pilot demonstration projects, they are able to employ a series of information technologies, as well as to obtain a strand of support from institutions. Therefore, does smart manufacturing pilot demonstration projects help to increase firms’ green innovation？

An emerging body of studies focus on the driving role of the digital economy in the upgrading of manufacturing industry [5]. However, few studies examined the impact of smart manufacturing-related policies on firms' green innovation. Though a few studies have explored how firms' digital transformation affects green production behaviors [6,7], it is important to note that corporate green production practices based on monitoring pollution levels are not the same as green innovation. Moreover, though several studies discussed on the impact of smart manufacturing on firms’ green innovation, it is also important to note that the impact of smart manufacturing is different from the impact of smart manufacturing demonstration projects, because firms listed in smart manufacturing demonstration projects not only realize digital transformation but also obtain supports from institutions.

In this context, using data from Chinese A-share listed companies between 2010 and 2020 and random forest method, this paper fills this gap by examining the impact of smart manufacturing demonstration projects on green innovation of firms. The principal contributions of this paper are as follows. First, the existing literature focuses primarily on the impact of smart manufacturing on the performance of firms. Although several studies have demonstrated its influence on green innovation, few descripted its internal mechanism in detail. As a complement to existing studies, this study examines the internal mechanism based on micro-data and concluded that the smart manufacturing demonstration projects promoted green innovation through enhancements in information processing capacities and innovation efficiency, as well as public attention increasement and signal effect. Second, in terms of methodology, previous studies have generally used fixed-effects panel data models to regress the relationship between firm performance and smart manufacturing demonstration projects. According to our knowledge, the predetermined relationship influences the effectiveness of the estimated results. Therefore, in this paper, we employ the random forest method, which can effectively address the interference problem and endogeneity issues associated with variable selection, and thus provide a more accurate assessment of the causal relationship between treatment variables and outcome variables [8].

The remainder of the paper is structured as follows. In Section 2, a literature review is conducted and research hypotheses are developed. The research design is described in Section 3. In Section 4, empirical results are presented. In Section 5, the results of the mechanism analysis are presented. In Section 6, discussions are developed. In the final section of the paper, the paper is concluded.

## 2 Research background and theory hypotheses

### 2.1 Research background

In recent years, a new round of technological and industrial revolution is emerging, represented by smart manufacturing. Many countries have begun to accelerate the adoption of smart manufacturing, including the Industrial Internet in the United States, Industry 4.0 in Germany, Horizon 2020 in the European Union and Japan's robotics strategy. In China, governments have issued “Made in China 2025” project. In 2016, the Ministry of Industry and Information Technology and the Ministry of Finance identified smart manufacturing as part of the manufacturing development strategic goals. Smart manufacturing helps to provide new support for green transformation and the realization of “dual carbon” goals. The Smart Manufacturing Pilot Demonstration Action Implementation Plan promulgated by the Ministry of Industry and Information Technology also proposes to promote the development of industrial low-carbon transformation through smart manufacturing, it is of great practical significance to study the impact of smart manufacturing on the green innovation of enterprises based on this policy.

### 2.2 Research hypotheses development

Following the “tragedy of the commons” theory, the environment is a public resource that is competitive and non-excludable [9]. In the absence of effective supervision, the “tragedy of the commons” is likely to occur, resulting in market failure. A market failure is widely regarded as evidence that political and government intervention is necessary [10]. Typically, the government promulgates regulations or incentives to correct market failure and encourage enterprises to optimize their production chains, thereby reducing environmental pollution, and making effective use of public resources. Likewise, green technology innovation suffers from problems associated with market failure, which require government legislation or incentives to ensure a competitive market environment. The goal of intelligent manufacturing demonstration projects is to promote the intelligent transformation and green development of enterprises, using green development orientation and incentives like tax exemptions and financial subsidies, to promote the intelligent transformation and green development of enterprises. Through the integration of advanced information technology and manufacturing technology, intelligent manufacturing facilitates intelligent transformation and data-based production within the manufacturing industry, strengthens the enterprise's proactive awareness of green innovation, reshapes the paradigm of green technology innovation, and thus affects the enterprise's capacity to innovate in green technology.

Enterprises participating in intelligent manufacturing demonstration projects will increase the construction of hardware and software such as information technology and improve the level of intelligence to promote green innovation. On the one hand, the introduction of intelligent equipment and information technology to improve the level of enterprise information technology is helpful to realize the collaborative sharing of information resources among enterprise departments and improve information processing ability. The improvement of information processing ability helps enterprises innovate market analysis ability, improve market green product demand forecasting and production decision-making ability, and increase green innovation to meet the market demand for green products. On the other hand, big data and digital platforms help enterprises control the whole process of production and research and development, achieve controllable green research and innovation process, and improve the efficiency of green innovation. In addition, the use of digital platforms promotes the integration and sharing of information resources among enterprises, realizes the flow and sharing of green innovation resources among enterprises, and improves the allocation efficiency of external green innovation resources. Therefore, the intelligent manufacturing demonstration project enables the enterprise informatization level, the improvement of information processing capacity and the improvement of innovation efficiency make green innovation oriented by market demand, and then encourage enterprises to implement green innovation. Based on the above discussion, the first research hypothesis is proposed as follows.

H1 Smart manufacturing demonstration projects promote firms' green innovation through information processing capability enhancement effect and innovation efficiency improvement effect.

In addition to the direct effects of information processing capabilities and green innovation efficiency, smart manufacturing demonstration projects affect green innovation through indirect effects. First of all, enterprises that enter smart manufacturing demonstration projects receive more public attention. Increased public awareness can help motivate companies to improve their environmental performance, and Xiang believes that high public awareness will force companies to increase green innovation [11]. Secondly, enterprises entering smart manufacturing demonstration projects are more likely to obtain financial support from financial institutions. Specifically, being included in smart manufacturing demonstration projects sends a signal to the government that these companies are strong and that local governments will increase support for political performance purposes. In addition, entering the smart manufacturing demonstration project can send a signal to the society of government certification to attract investors' attention [12]. The government certification signal can ease the information asymmetry between banks and enterprises, thereby increasing the opportunity for enterprises to obtain external funding. The positive signal effect can help enterprises obtain capital expenditure, reduce the financing constraint of enterprises, and promote the green innovation of enterprises. Accordingly, the second research hypothesis is proposed as follows.

H2 Smart manufacturing demonstration projects promote green innovation through the public attention enhancement effect and signal effect.

The impact of manufacturing demonstration projects on corporate green innovation varies by industry. Firstly, labor-intensive industries focus more on cost and production scale, and less on green R&D investment and green innovation efficiency [13]. Consequently, this type of industry mostly replaces simple and repetitive production activities with smart manufacturing, so it's hard to have a big impact on green innovation. In addition, capital-intensive industries rely heavily on advanced equipment and machinery. Enterprises that implement smart manufacturing demonstration projects benefit not only from intelligent machinery and equipment, but also from government subsidies and external investment assistance. Therefore, smart manufacturing demonstration projects can promote capital-intensive enterprises to achieve green innovation outputs by providing advanced equipment and financial support [14,15]. Finally, while technology-intensive industries have plenty of advantages, like advanced production equipment, innovation efficiency, etc. [16], because their technological level is close to cutting-edge technology, there's not much room for enterprises to innovate. Without major breakthroughs in basic research, it will be difficult to improve the level of green innovation only by smart manufacturing [17]. Accordingly, this paper proposes.

H3 The positive effect of smart manufacturing demonstration projects on green innovation is pronounced for capital-intensive firms.

Firstly, human resources are the main bottleneck for smart manufacturing [18]. There are huge differences in human resources in different regions of China, so the green innovation effect in different regions of China will be heterogeneous [19]. Because of its geographical and economic advantages, the eastern part of China is very attractive to human resources [20,21], which can break the bottlenecks that stop enterprises from manufacturing intelligently, and then promote green innovation more effectively. Human resource shortages in western and central regions will limit the green innovation effect of intelligent manufacturing. In addition, enterprise smart manufacturing needs big data centers ‘support. As a result of its climate and large land mass, the western region is a good place to build big data centers, resulting in a better information infrastructure for enterprise intelligent manufacturing and ultimately promoting it [22]. Lastly, the central region lacks human resources and new infrastructure. In contrast, because of the high number of state-owned enterprises, enterprises don't have enough innovation vitality, so the green innovation effect of smart manufacturing is low. Accordingly, the following research hypotheses are put forward.

H4 The positive effect of smart manufacturing demonstration projects on green innovation is pronounced for firms located in eastern and western regions.

## 3 Data, variables, and methodology

### 3.1 Data

In this paper, we use Chinese A-share listed companies from 2010 to 2020 as the research sample. Our data are from two databases. The first one is the China Stock Market & Accounting Research (CSMAR) database which provides firm-level financial data and general information. The second one is the patent database of the State Intellectual Property Office which provides patent data. In order to obtain firm-level green patent data, we also employ the “Green List of International Patent Classification” issued by the World Intellectual Property Organization.

After merging the two databases based on stock code, we further exclude observations satisfying one of the following criteria: (i) financial industry and insurance industry; (ii) main variables’ value missing or abnormal. Finally, the sample includes 31,292 observations, where 114 enterprises in the treatment group and 4016 enterprises in the control group.

#### 3.1.1 Variables

##### 3.1.1.1 Green innovation

Based on existing studies, we measure the green innovation of enterprises by using the number of green patent grants [23]. Specifically, the sum of the number of green invention patents granted and the number of green utility model patents granted is used to measure firm-level green innovation (total). In addition, we use the number of green invention patents granted to measure corporate substantive innovation (event) and the number of green utility model patents granted to measure corporate strategic innovation (utility). To eliminate the problem of right-skewed distribution, the logarithm of the number of green patents granted adding one is used in this paper.

#### 3.1.2 Treatment variable

According to the “Notice of the Ministry of Industry and Information Technology on the Announcement of Smart Manufacturing Demonstration Projects” (2015–2018) issued by the Ministry of Industry and Information Technology，we manually identify the companies selected for smart manufacturing demonstration projects. If it belongs to smart manufacturing firms, then the value of the treatment variable is “1”, otherwise “0”.

#### 3.1.3 Control variables

Based on Amore and Bennedsen [24], the following variables are controlled in this paper: (i) Firm size. The larger the firm size, the higher the innovation output [25], so we use the total *assets* and the number of *employees* to measure the size of the enterprise. (ii) Firm age. The longer established enterprises can easily grasp the market information and their innovation consciousness is stronger [26], so this paper uses the logarithm of the statistical year minus established year plus one to measure the *firm age*. In addition, this paper also controls for firm value (*Tobin's Q)*, leverage (*debt*), cash flow (*cash*), the percentage of shares held by a firm's top ten shareholders (*share*), and time, region, and industry fixed effects. The descriptive results are shown in Table 1.

**Table 1:** Descriptive Statistics.

Variable	Indicator Meaning	Observations	Mean	SD	Min	Max
*total*	Logarithm of total number of green granted patents	31292	0.55	0.93	0.00	6.79
*invent*	Logarithm of the number of green invention licenses	31292	0.21	0.57	0.00	6.14
*utility*	Logarithm of the number of green utility model authorizations	31292	0.48	0.85	0.00	6.27
*asset*	Log of total business assets	31292	22.09	1.35	13.76	28.64
*worker*	Logarithm of the number of employees in the company	31292	7.57	1.34	0.69	13.22
*age*	Logarithm of corporate age	31292	2.85	0.36	0.00	4.14
*Tobin's Q*	Total market capitalization/assets	31292	2.33	12.04	0.67	1752.71
*debt*	Gearing ratio	31292	0.45	1.14	−0.20	178.35
*cash*	Total cash/assets	31292	0.19	0.15	0.00	0.99
*share*	Shareholding ratio of top ten shareholders	31292	0.59	0.16	0.04	1.00

### 3.2 Methodology

Public policy evaluation is integral to the effective implementation of public policies. Most early public policy evaluations were based on theoretical research, primarily using qualitative and simple quantitative methods [27]. As social sciences continue to develop, econometrics has gradually become a mainstream method for evaluating public policy. Today, mainstream econometric methods for evaluating public policy include regression correction methods (RA), matching methods (Matching), instrumental variables methods (IV), double difference methods (DID), and regression discontinuity methods (RDD).

Green innovation has been a popular direction in economics research in recent years. Some studies have utilized the difference-in-differences method (DID) to study environmental equity transactions, including Weber and Neuhoff [28], Xie et al. [29], Zhang et al. [23], and Carrión-Flores et al. [30]. According to the study of the impact of public policies, such as regulations, on green innovation, environmental regulations can encourage corporate innovation. The DID method requires that the control group and the treatment group have a parallel trend in the early stages of the experiment. According to Amore [24], Kock et al. [31], and Krueger et al. [32], the governance structure and environmental quality management system were studied using regression adjustment (RA) and instrumental variable methods (IV). According to the research results, stakeholder pressure has a positive effect on corporate green innovation. Data quality and weak instrumental variables may adversely affect the validity of these two regression methods. The regression discontinuity method (RDD) was used by Mato examine how green credit policies influence the green innovation of listed companies and found that green credit policies promote corporate green innovation by reducing financing constraints [33]. For the RDD, a certain threshold value separates the non-treatment group from the treatment group. Setting the critical interval of the threshold value will have an impact on the results of the estimation.

Mainstream public policy measurement methods have problems with matching variable selection interference and endogeneity, and cannot truly and effectively evaluate policy effects. To figure out the impact of smart manufacturing demonstration projects on firms’ green innovation, we employ random forest methods. Unlike the traditional regression methods(RA、PSM-DID、RDD)based on the assumption of predetermined relationships, the random forest method is not easily disturbed by the selection of control variables and can reduce the dependence of the estimation results on subjective selection criteria, so it can reflect the causal relationship between the independent and dependent variables. The specific estimation method is as follows:

Assume that the samples (*X*_*i*_, *Y*_*i*_, *W*_*i*_) are independently and identically distributed, where *i* = 1, 2, 3, …, n, *X*_*i*_ consists of control variables, *Y*_*i*_ ∈ *R* represents the response outcome, and *W*_*i*_ is the treatment variable. There are two response outcomes: *Y*_*i*_^*(0)*^ represents the response outcome where *i* does not receive processing, and *Y*_*i*_^*(1)*^ represents the response outcome where *i* receives processing. Then, the result of the treatment at $X_{i}$ is shown in equation (1):

(1) $$\tau \left(x\right)=Ε\left[Y_{i}^{\left(1\right)}-Y_{i}^{\left(0\right)}|X_{i}=x\right]$$

In order to estimate τ(x) effectively, we assume that the treatment variable *W*_*i*_ is independent of the response value *Y*_*i*_, As shown in equation (2):

(2) $$\left\{Y_{i}^{\left(0\right)},Y_{i}^{\left(1\right)}\right\}⊥W_{i}|X_{i}$$

We further assume that the observations of the nearest neighbors in *x*-space are as a random distribution, which in turn yields equation (3):

(3) $$\tau \left(x\right)=Ε\left[Y_{i}\left(\frac{W_{i}}{e\left(x\right)}-\frac{1-W_{i}}{1-e\left(x\right)}\right)|X_{i}=x\right],e\left(x\right)=Ε\left[W_{i}|X_{i}=x\right]\frac{x-\mu }{\sigma }$$

Where *e(x)* represents the probability of receiving treatment at *x.* By estimating *e(x), the* unbiased estimator of τ(x) can then be solved.

Based on existing studies^8^, we can achieve consistent estimation by using equation (2): first, only independent samples (*X*_*i*_, *Y*_*i*_) are observed and partitioned into a causal tree of *L leaf* nodes by building a CART regression tree; second, given a demonstration point *x,* equation (4) is evaluated by identifying the leaf node *L*(*x*) as follows:

(4) $$\hat{\mu }\left(x\right)=\frac{1}{\left|\left\{i:X_{i}\in L\left(x\right)\right\}\right|}\sum_{\left\{i:X_{i}\in L\left(x\right)\right\}}Y_{i}$$

If L(*x*) is sufficiently small, the data can be assigned to each leaf *L.* The observations *X*_*i*_ distributed in each leaf are so close to the random trial distribution that the response values *Y*_*i*_ within the leaf nodes are essentially identically distributed. The final average treatment effect to be estimated is in equation (5):

(5) $$\hat{\tau }\left(x\right)=\frac{1}{\left|\left\{i:W_{i}=1,X_{i}\in L\left(x\right)\right\}\right|}\sum_{\left\{i:W_{i}=1,X_{i}\in L\left(x\right)\right\}}Y_{i}-\frac{1}{\left|\left\{i:W_{i}=0,X_{i}\in L\left(x\right)\right\}\right|}\sum_{\left\{i:W_{i}=0,X_{i}\in L\left(x\right)\right\}}Y_{i}$$

Inspired by Acemoglu and Finkelstein [34], the treatment group was defined as the firms selected for the smart manufacturing demonstration projects and the control group as the non-selected firms. The treatment effects that need to be estimated when applying the random forest approach to causal inference are shown in equation (6):

(6) $$\hat{\tau }\left(x\right)=\frac{1}{\left|\left\{i:W_{i}=1,t_{i}=1,X_{i}\in L\left(x\right)\right\}\right|}\sum_{\left\{i:W_{i}=1,t_{i}=1,X_{i}\in L\left(x\right)\right\}}Y_{i}-\frac{1}{\left|\left\{i:W_{i}=0,t_{i}=0,X_{i}\in L\left(x\right)\right\}\right|}\sum_{\left\{i:W_{i}=0,t_{i}=0,X_{i}\in L\left(x\right)\right\}}Y_{i}$$

Where, $\hat{\tau }\left(x\right)$ denotes the treatment result of the smart manufacturing demonstration projects; *W*_*i*_ = 1 is the treatment group, *i.e.*, the enterprises selected for the smart manufacturing demonstration projects, and *W*_*i*_ = 0 is the control group, *i.e.,* the enterprises not selected for the smart manufacturing demonstration projects. *t*_*i*_ is the time dummy variable, before the implementation of the smart manufacturing demonstration projects (2014 and before) such that *t*_*i*_ = *0*, and after the implementation of the smart manufacturing demonstration projects (2015 and after) such that *t = 1*).

In this paper, 70% of the data in the original dataset are randomly selected to generate the training dataset, and the remaining 30% are used to form the corresponding test dataset to improve the representativeness of the samples as much as possible.

## 4 Empirical results

### 4.1 Baseline results

Table 2 reports the baseline results. In addition to the control variables, year and region fixed effects are added to column (1), and industry fixed effects are further added to column (2). The results show that the coefficient is significantly positive at the level of 1%, that is, after the implementation of intelligent manufacturing demonstration projects, the average number of green innovation patents of enterprises increases by 0.269%, indicating that intelligent manufacturing demonstration projects can promote green innovation of enterprises. In columns (3) and (4), green invention patents and green utility model patents are used to measure corporate green innovation, respectively. The results show that smart manufacturing demonstration projects can promote both substantive green innovation and strategic green innovation.

**Table 2:** Baseline results.

	total	total	invent	utility
Treatment Effect	0.3753***	0.2687***	0.2766***	0.2099***
	(0.0694)	(0.0633)	(0.0527)	(0.0596)
Observations	31292	31292	31292	31292
Control variables	Yes	Yes	Yes	Yes
Year fixed effect	Yes	Yes	Yes	Yes
Region fixed effect	Yes	Yes	Yes	Yes
Industry fixed effect	No	Yes	Yes	Yes

Note: *, ** and *** denote 10%, 5% and 1% significance levels, respectively. The same in the following tables.

**Fig. 1:**
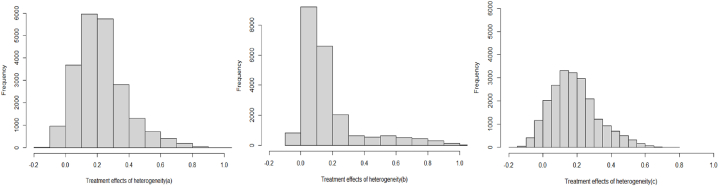
Spectrum of Random Forest Estimation Results Note: The explanatory variables in (a)–(c) are the total number of green patents granted, the number of green invention patents granted, and the number of green utility model patents granted, respectively.

Fig. 1 shows that the distribution probability of the positive treatment effect is greater than that of the negative treatment effect, and the average treatment effect is positive, further indicating that the smart manufacturing demonstration projects promote firms’ green innovation.

In addition, according to the distribution probabilities of the treatment effects in (b) and (c) of Fig. 1, the treatment effect of smart manufacturing demonstration projects on substantive green innovation is concentrated at a higher frequency to the right of the zero, indicating that smart manufacturing demonstration projects promote substantive green innovation of enterprises greater than strategic green innovation.

### 4.2 Robustness test

#### 4.2.1 New measurement

With reference to the research of Liu and Dong [35], this paper adopts the number of green patent applications to measure the green innovation of enterprises, the number of green invention patents to measure the substantive green innovation of enterprises, and the number of green utility model patents to measure the strategic innovation of enterprises, and adopts the number plus one to take the natural logarithm, and the number of green patents to measure the green innovation of enterprises. The results are shown in Table 3, and the coefficients shown in the results are significantly positive, consistent with the benchmark results.

**Table 3:** Mechanism test: Information processing capacity improvement effect.

Variables	*info*	*info*	*effi*	*effi*
Treatment Effect	0.6634***	0.6798***	0.0369**	0.0349*
	(0.0734)	(0.0709)	(0.0182)	(0.0183)
Observations	25001	25001	16108	16108
Control variables	Yes	Yes	Yes	Yes
Year fixed effect	Yes	Yes	Yes	Yes
Region fixed effect	Yes	Yes	Yes	Yes
Industry fixed effect	NO	Yes	NO	Yes

#### 4.2.2 New sample

On July 19, 2010, the National Development and Reform Commission issued the Notice on the Demonstration of Low-Carbon Provinces, Regions, and Cities to demonstrate low-carbon cities in five provinces and eight cities, and then established 29 and 45 demonstration cities in 2012 and 2017, respectively. The demonstration work of low-carbon cities might affect firms’ green innovation. To exclude the impact of low-carbon city policies, we re-estimate the treatment effect by excluding the enterprises in low-carbon demonstration cities. The results in Table 4 confirm that our baseline results are robust.

**Table 4:** Mechanism test: Public concern and environmental information disclosure.

Variables	*search*	*search*	*envir*	*envir*
Treatment Effect	0.1234***	0.1436***	0.1480***	0.1629***
	(0.0283)	(0.0294)	(0.0321)	(0.0319)
Observations	27268	27268	30988	30988
Control variables	Yes	Yes	Yes	Yes
Year fixed effect	Yes	Yes	Yes	Yes
Region fixed effect	Yes	Yes	Yes	Yes
Industry fixed effect	No	Yes	No	Yes

#### 4.2.3 New method

In order to obtain robustness results. We further adopt Bayesian Additive Regression Trees (BART), Matching, and Propensity Score Matching (PSM) approaches to estimate the treatment effect. The results are reported in Table 5. The coeffects of treatment variable are all significantly positive at 1% significance level, indicating that smart manufacturing demonstration projects increase the green innovation of firms.

**Table 5:** Mechanism test: Signal effect.

Variables	(1)	(2)	(3)	(4)
Treatment Effect	0.3753***	0.3540***	−0.1241*	−0.1417**
	(0.0565)	(0.0552)	(0.0707)	(0.0717)
Observations	30134	30134	31292	31292
Control variables	Yes	Yes	Yes	Yes
Year fixed effect	Yes	Yes	Yes	Yes
Region fixed effect	Yes	Yes	Yes	Yes
Industry fixed effect	No	Yes	No	Yes

#### 4.2.4 Different batches

In 2015, 2016, 2017 and 2018, the Ministry of Industry and Information Technology announced four batches of smart manufacturing demonstration companies. The policies of different batches of demonstration companies are the same. To test the robustness of the impact of smart manufacturing on corporate green innovation, this paper examines several batches of demonstration enterprises, with empirical results shown in Table 6. It has been determined that the intelligent manufacturing demonstration project has had a significant positive impact on the green innovation of the four batches of demonstration enterprises, i.e., the intelligent manufacturing demonstration project has promoted green innovation in enterprises.

#### 4.2.5 Heterogeneous timing DID

In this paper, the heterogeneous timing DID method is used to estimate the impact of smart manufacturing demonstration projects on green innovation. Table 7 summarizes the results. Smart manufacturing demonstration projects have a significant positive impact on green innovation, according to the results.

## 5 Internal mechanism tests and heterogeneity checks

### 5.1 Internal mechanism tests

To testify the information processing capabilities enhancement effect, we use the logarithm of the value of software capital and hardware capital inputs to measure firms' information processing capability (*info*), where hardware capital is calculated by using enterprises' electronic equipment, communication equipment and computers and auxiliary equipment, and software capital is estimated by using enterprise. The corresponding data are obtained from the RESET financial database. Columns 1 and 2 of Table 3 report the impact of smart manufacturing demonstration projects on firms’ information processing capability. The results show that the treatment effect is significantly positive, indicating that the smart manufacturing demonstration projects improve information processing capability.

Next, we test the innovation efficiency improvement effect. Firstly, we use R&D input and R&D personnel as input factors and the number of green patents granted as output variables to calculate firms' green innovation efficiency (*effi*) with the help of the DEA-Malmquist index. Based on this method, we calculate total innovation efficiency to reflect the efficiency of enterprises to invest R&D expenses and R&D personnel to implement innovation at the current scale, pure technical efficiency to measure enterprises’ green innovation efficiency, and scale efficiency. The results in columns (3) and (4) of Table 3 show that smart manufacturing demonstration projects improve innovation efficiency.

Referring to we select the index of enterprise web search (*SEARCH*) as a proxy variable for social public concern. We select the index of enterprise environmental information disclosure (*ENVIR*) as a proxy variable for environmental information disclosure quality.

The results in Table 4 show that smart manufacturing demonstration projects significantly increase the public attention of selected enterprises and the quality of environmental information disclosure of enterprises, while higher social public attention and quality of environmental information disclosure will bring more pressure on enterprises for green transformation, thus forcing green innovation.

We use government subsidies (*sub*) received by enterprises to measure the strength of government policy support, and refer to the method of Kaplan and Zingales [36] to construct the *KZ* index to measure the support from financial institutions. The *KZ* index reflects the financing constraints faced by enterprises∗, the larger the value, the stronger the financing constraints.

The results in Table 5 show that the smart manufacturing demonstration projects improve the government subsidies received by the selected enterprises and alleviate the financing constraints, thus proving that there is a signal effect of the smart manufacturing demonstration projects， which can help the selected enterprises to obtain more government funds and financing support from financial institutions to promote green innovation.

### 5.2 Heterogeneity checks

#### 5.2.1 Industry heterogeneity

Based on the National Economic Classification of Industries, all industries are classified into three categories: labor-intensive industries, capital-intensive industries and technology-intensive industries using cluster analysis by calculating the proportion of fixed assets and the proportion of R&D expenditures in each industry. The results are shown in Table 11. It indicates that the positive effect of the smart manufacturing demonstration projects on firms’ green innovation is pronounced for firms in capital-intensive industries, which may be because corporate smart manufacturing and green innovation require abundant capital support [37].

#### 5.2.2 Regional heterogeneity

The external environment might influence the effect of manufacturing demonstration projects on green innovation. Accordingly, based on firms’ regional information, we divide the sample into three subsamples: eastern, central, and western regions. The results in Table 12 show that the treatment effect is significantly positive in the eastern and western regions and insignificant in the central region. The result shows that for firms located in the central region, the smart manufacturing demonstration projects have no effect on green innovation. This may be because the central region is in the development stage of undertaking the transfer of industries from the east, with a continuous turnover of new and old industries, which affects the positive role of smart manufacturing demonstration projects.

#### 5.2.3 Corporate cash flow heterogeneity

Technology innovation requires a significant amount of capital investment on the part of enterprises. The internal cash flow of enterprises has become one of the most important factors in determining technological innovation in the face of external financing constraints. Accordingly, smart manufacturing has a different impact on corporate green innovation for companies with different levels of cash holdings. In accordance with Opler et al. [38], the ratio of corporate cash to total assets is used to measure corporate cash holding levels, and the regression is divided into two groups according to the median annual level of corporate cash holdings. Table 13 presents the results. Compared with companies with high cash holding levels, the estimated coefficients of companies with low cash holding levels are larger. The need for external financing is greater for companies that lack internal cash. As a result, smart manufacturing brings government subsidies in addition to external financing channels that can effectively satisfy corporate financing needs, thereby stimulating corporate green innovation.

## 6 Discussion

Intelligent manufacturing represents the key to increasing the competitiveness of the manufacturing industry and achieving high-quality development as the leading force in the new round of technological revolution. Studying the impact of intelligent manufacturing demonstration projects on corporate green innovation can enhance research related to intelligent manufacturing and make recommendations for policy development. The purpose of this paper is to explore how smart manufacturing projects can improve corporate green innovation. There are four mechanisms through which smart manufacturing demonstration projects affect corporate green innovation: corporate information processing capabilities, innovation efficiency, social public attention, and signaling effects.

There are several contributions of this article: firstly, it enriches research in the field of intelligent manufacturing. Intelligent manufacturing research has traditionally focused primarily on corporate performance [4,39], while green innovation research has been primarily based on macro data [40], lacking microscopic mechanisms. Research on green innovation primarily focuses on environmental regulation [41,42], green credit [43], and digital transformation [44]. Few articles study intelligent manufacturing and corporate green innovation, so this article fills that gap. Second, by comparing conventional measurement methods with predetermined relationship assumptions, this article identifies the cause and effect between smart manufacturing demonstration projects and corporate green innovation using the random forest method in machine learning. By reducing interference and endogeneity problems in matching variable selection, relationships can be used to assess the true relationship more objectively and accurately between variables [8]. The findings of this article confirm that smart manufacturing demonstration projects improve corporate green innovation by improving information processing capabilities, improving innovation efficiency, attracting social attention, and increasing financial subsidies. Policy suggestions are provided to assist the government in formulating policies and achieving carbon neutrality.

While the sample for the smart manufacturing demonstration project is limited to China's A-share listed companies, some unlisted companies have also been selected for the project. The research on smart manufacturing demonstration projects can be improved by obtaining data from unlisted companies in the future. In addition, a number of countries around the world have proposed plans for deploying intelligent manufacturing. Since national conditions and levels of economic development differ, there are differences in the policy measures and expected outcomes of implementing intelligent manufacturing demonstration projects. It may be difficult to apply this research model directly. It is necessary to discuss how to study other countries in more detail. Lastly, further research and improvement are necessary to determine whether smart manufacturing demonstration projects have long-term impacts, whether companies focus on long-term development rather than short-term green innovations in response to policy implementation, and what the long-term effects are after policy implementation.

## 7 Conclusion

Smart manufacturing is the core strategy for China's manufacturing industry to bring into play its latecomer advantage to achieve transformation and catch-up. This paper identifies the impact of smart manufacturing demonstration projects on the green innovation of firms by using random forest approaches. The main results are as follows. Firstly, smart manufacturing demonstration projects significantly promote enterprise green innovation and have a greater effect on substantive innovation than strategic innovation. The baseline results are supported by a series of robustness checks. Second, the internal mechanism tests show that the smart manufacturing demonstration projects promote green innovation of firms through the information processing capability enhancement effect, innovation efficiency enhancement effect, public attention enhancement effect, and signal effect. Third, the heterogeneity checks show that the positive effect of smart manufacturing demonstration projects on green innovation of firms is pronounced for capital-intensive firms, and firms in western and eastern regions.

The above findings have several primary policy implications. First, from the perspective of policymakers, they should provide more advantages for manufacturing firms to realize smart manufacturing. Moreover, policymakers also should improve public attention and government subsidies to promote firms’ green innovation. Second, from the perspective of manufacturing firms, they should take advantage of information technologies, improving their information processing capabilities and innovation efficiency. Third, considering the heterogeneity effects of smart manufacturing demonstration projects on green innovation of firms, local governments should employ different projects for firms in different industries and for firms in different regions.

Despite several contributions, there are several limitations. Firstly, we only focus on the A-share listed firms due to data availability. More samples of companies to verify in the future are very interesting. Secondly, for the heterogeneity effects of smart manufacturing demonstration projects on green innovation of firms, this paper only takes industry heterogeneity and location differences into account. Further research should consider more heterogeneous if the approaches are available.

## Funding statement

Pro. Jun Yang was supported by The 10.13039/501100012456National Social Science Fund of China [23BJY124].

## Data availability statement

All data required to support the result and conclusion of study are available on request.

## CRediT authorship contribution statement

**Jun Yang:** Formal analysis, Data curation. **Weihao Wang:** Visualization, Validation, Software. **Chunheng Fu:** Validation, Supervision. **Xiaohui Xu:** Conceptualization. **Qiuzhen Li:** Writing – review & editing.

## Declaration of competing interest

The authors declare the following financial interests/personal relationships which may be considered as potential competing interests:Jun Yang reports financial support was provided by 10.13039/501100012456National Social Science Foundation of China(23BJY124). If there are other authors, they declare that they have no known competing financial interests or personal relationships that could have appeared to influence the work reported in this paper.

## Figures and Tables

**Table 3:** Robustness Test: New measurement

Variables	*atotal*	*ainvent*	*autility*	*total*
Treatment Effect	0.3031***	0.3118***	0.2221***	7.3500***
	(0.0699)	(0.0645)	(0.0603)	(2.0300)
Observations	31292	31292	31292	31292
Control variables	Yes	Yes	Yes	Yes
Year fixed effect	Yes	Yes	Yes	Yes
Region fixed effect	Yes	Yes	Yes	Yes
Industry fixed effect	Yes	Yes	Yes	Yes

**Table 4:** Robustness Test: Eliminate Low-carbon Cities

Variables	(1) *total*	(2) *invent*	(3) *Utility*
Treatment Effect	0.3043***	0.3064***	0.1921***
	(0.0658)	(0.0602)	(0.0603)
Observations	26453	26453	26453
Control variables	Yes	Yes	Yes
Year fixed effect	Yes	Yes	Yes
Region fixed effect	Yes	Yes	Yes
Industry fixed effect	Yes	Yes	Yes

**Table 5:** Robustness Test: New methods

	BART	Matching	PSM
Treatment Effect	0.2687***	0.2766***	0.2099***
	(0.0633)	(0.0527)	(0.0596)
Observations	31292	31292	31292
Control variables	Yes	Yes	Yes
Year fixed effect	Yes	Yes	Yes
Region fixed effect	Yes	Yes	Yes
Industry fixed effect	Yes	Yes	Yes

**Table 6:** Robustness tests：Different batches

Variables	(1)	(2)	(3)	(4)
Treatment Effect	0.2510**	0.4940***	0.2135**	0.1945*
	(0.1120)	(0.1310)	(0.0983)	(0.0990)
Observations	30451	30484	30507	30531
Control variables	Yes	Yes	Yes	Yes
Year fixed effect	Yes	Yes	Yes	Yes
Region fixed effect	Yes	Yes	Yes	Yes
Industry fixed effect	Yes	Yes	Yes	Yes

**Table 7:** Robustness tests Heterogeneous timing DID

Variables	(1) lntotal	(2) lntotal	(3) lntotal
W	0.7000***	0.3330***	0.1400***
	(0.0617)	(0.0550)	(0.0472)
TbinQ		0.0024***	0.0010***
		(0.0008)	(0.0003)
share		−0.3260***	−0.0865*
		(0.0312)	(0.0487)
lnasset		0.2990***	0.1980***
		(0.0050)	(0.0083)
lnage		−0.1300***	0.1580***
		(0.0151)	(0.0521)
Cash		0.0509*	0.0286
		(0.0300)	(0.0317)
debt		0.0054	−0.0059
		(0.0055)	(0.0049)
FE	No	No	Yes
Year fixed effect	Yes	Yes	Yes
Region fixed effect	Yes	Yes	Yes
Industry fixed effect	Yes	Yes	Yes
Observations	31,292	31,292	30,894
R-squared	0.218	0.353	0.729

**Table 11:** Heterogeneity Test: Industry heterogeneity

Variables	(1) Labor-intensive	(2) Technology-intensive	(3) Capital-intensive
Treatment Effect	−0.0184	0.0655	0.2178***
	(0.0789)	(0.0851)	(0.0902)
Observations	8369	12296	10627
Control variables	Yes	Yes	Yes
Year fixed effect	Yes	Yes	Yes
Region fixed effect	Yes	Yes	Yes
Industry fixed effect	Yes	Yes	Yes

**Table 12:** Heterogeneity Test: Regional heterogeneity

Variables	(1) Eastern	(2) Central	(3) Western
Treatment Effect	0.3563***	0.1790	0.3590**
	(0.0737)	(0.1140)	(0.1760)
Observations	21876	5099	4314
Control variables	Yes	Yes	Yes
Year fixed effect	Yes	Yes	Yes
Region fixed effect	Yes	Yes	Yes
Industry fixed effect	Yes	Yes	Yes

**Table 13:** Heterogeneity test：Corporate cash flow heterogeneity

Variables	(1) low cash flow	(2) high cash flow
Treatment Effect	0.5730***	0.1678**
	(0.1060)	(0.0789)
Observations	21876	15649
Control variables	Yes	Yes
Year fixed effect	Yes	Yes
Region fixed effect	Yes	Yes
Industry fixed effect	Yes	Yes

## Footnotes

∗ Due to the lack of data on green credit for listed companies, only financing constraint indicators are used in this section for analysis.
